# Stem Cell Metabolism in Cancer and Healthy Tissues: Pyruvate in the Limelight

**DOI:** 10.3389/fphar.2017.00958

**Published:** 2018-01-04

**Authors:** Cyril Corbet

**Affiliations:** Pole of Pharmacology and Therapeutics, Institut de Recherche Expérimentale et Clinique, Université catholique de Louvain, Brussels, Belgium

**Keywords:** pyruvate, metabolism, stem cell, cancer, glycolysis, oxidative phosphorylation

## Abstract

Normal and cancer stem cells (CSCs) share the remarkable potential to self-renew and differentiate into many distinct cell types. Although most of the stem cells remain under quiescence to maintain their undifferentiated state, they can also undergo cell divisions as required to regulate tissue homeostasis. There is now a growing evidence that cell fate determination from stem cells implies a fine-tuned regulation of their energy balance and metabolic status. Stem cells can shift their metabolic substrate utilization, between glycolysis and mitochondrial oxidative metabolism, during specification and/or differentiation, as well as in order to adapt their microenvironmental niche. Pyruvate appears as a key metabolite since it is at the crossroads of cytoplasmic glycolysis and mitochondrial oxidative phosphorylation. This Review describes how metabolic reprogramming, focusing on pyruvate utilization, drives the fate of normal and CSCs by modulating their capacity for self-renewal, clonal expansion/differentiation, as well as metastatic potential and treatment resistance in cancer. This Review also explores potential therapeutic strategies to restore or manipulate stem cell function through the use of small molecules targeting the pyruvate metabolism.

## Introduction

Stem cells display the capacity for self-renewal, commitment to clonal expansion and multipotent differentiation (Morrison et al., 1997; Weissman et al., 2001), allowing them to drive development and control tissue homeostasis. The stem cell compartment is a largely quiescent (“slow-cycling”) cell subset where intermittent mitotic activity reduces stress damage. When activated, stem cells may exhibit different fates that include self-renewal and clonal expansion to maintain stem and progenitor cell pools, the latter then driving regeneration of functional tissues. The balance between self-renewal and clonal expansion/differentiation, which is critical to regulate the size of the stem cell populations, implies distinct metabolic requirements. Indeed, most proliferating stem cell populations exhibit a glycolytic metabolic program to support synthesis of cellular building blocks for sustaining cell growth (Simsek et al., 2010; Ito and Suda, 2014). During differentiation, this metabolic asset switches to a program of mitochondrial carbohydrate oxidation to generate ATP and reducing equivalents in a more efficient way (Berger et al., 2016; Rodriguez-Colman et al., 2017). Among the variety of energetic substrates, pyruvate has emerged as a major contributor for the control of stem cell homeostasis. While a relationship between pyruvate and progenitor/stem cell function was suggested more than 30 years ago (Dello Sbarba et al., 1987), pyruvate metabolism has lately gained attention in clinical oncology as small molecules targeting those specific metabolic pathways can affect cancer stem cell (CSC) proliferation and self-renewal capacities, and thereby offer new therapeutic perspectives.

### Glycolysis, OXPHOS, and Stem Cell Fate

**FIGURE 1:**
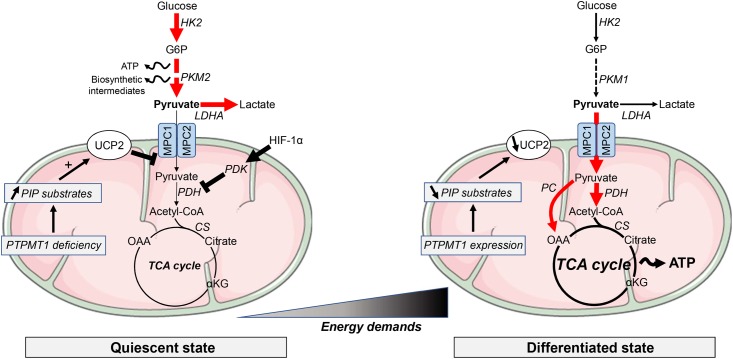
Metabolic requirements in pluripotent stem cells and differentiated cells. Most of the quiescent stem cell populations rely on glycolysis to provide ATP and building blocks necessary for the synthesis of lipids, proteins or nucleotides. Along differentiation, energy metabolism shifts toward mitochondrial OXPHOS to support the increasing energy demands. Glycolysis-associated enzymes, such as hexokinase (HK), pyruvate kinase (PK) and lactate dehydrogenase A (LDHA), are highly expressed in PSCs. Pyruvate import into mitochondria is repressed by high expression of uncoupling protein 2 (UCP2). UCP2 is upregulated by accumulation of phosphatidylinositol phosphate (PIP) substrates following the downregulation of the mitochondrial phosphatase PTPMT1. αKG, α-ketoglutarate; ATP, adenosine triphosphate; CS, citrate synthase; G6P, glucose 6-phosphate; HIF, hypoxia-inducible factor; MPC, mitochondrial pyruvate carrier; PDH, pyruvate dehydrogenase; PDK, PDH kinase; OAA, oxaloacetate. Elevated metabolic pathways are indicated with bold arrows.

Pyruvate is a central metabolite for glucose, lactate, lipids, and amino acids. From glucose, pyruvate is synthesized by the pyruvate kinase (PK) in the last step of glycolysis. Pyruvate can then follow alternative metabolic routes. Pyruvate reduction into lactate *via* lactate dehydrogenase A (LDHA) facilitates the rapid recycling of NAD^+^ necessary to maintain a high glycolytic rate. In the oxidative route, pyruvate is first imported into the mitochondrial matrix where it can fuel the tricarboxylic acid (TCA) cycle either by feeding into mitochondrial acetyl-CoA synthesis *via* pyruvate dehydrogenase (PDH) and citrate synthase or by generating oxaloacetate through pyruvate carboxylase activity (**Figure 1**). Pyruvate can also be converted, in both cytosolic and mitochondrial compartments, into alanine or malate through alanine aminotransferase (ALT) or malic enzyme (ME) activity, respectively. The fate of pyruvate depends on many factors, one of which being oxygen availability. Most of the stem cell populations, including hematopoietic stem cells (HSCs), mesenchymal stem cells (MSCs) and neural stem cells (NSCs), reside in hypoxic niches *in vivo* and thus have to prevent oxidative stress-induced senescence and to preserve long-term self-renewal potential (Simsek et al., 2010; Suda et al., 2011). Many studies have reported that stem cells mostly rely on glycolysis, in a hypoxia-inducible factor 1α (HIF-1α)/pyruvate dehydrogenase kinase (PDK)-dependent manner, for energy production (Varum et al., 2011; Takubo et al., 2013) while a switch to mitochondrial respiration is necessary upon differentiation (Yu et al., 2013). Expressions of hexokinase 2 (HK2), LDHA and the specific isoform PKM2 were found to support the maintenance of the pools of HSCs and NSCs (Wang et al., 2014; Zheng et al., 2016). In the latter, loss of HK2 and LDHA, associated with a switch in *PK* gene splicing from PKM2 to PKM1 expression, is indispensable for the transition from glycolysis toward OXPHOS during cell differentiation, as constitutive expression of the enzymes led to neuronal cell death (Zheng et al., 2016). In HSCs, PKM2 depletion decreased glycolysis while promoting OXPHOS specifically in the primitive stem/progenitor cell population (Wang et al., 2014). Interestingly, the authors reported that this metabolic switch was also associated with decreased capacities of both acute and chronic myeloid leukemia models to develop *in vivo*.

Among the molecular components involved in this metabolic switch, PTEN-like mitochondrial phosphatase PTPMT1 was found to be required for mitochondrial respiration and differentiation of HSCs (Yu et al., 2013) (**Figure 1**). Indeed, PTPMT1 depletion resulted in hematopoietic failure in mice and a blockade in the differentiation of HSCs both *in vitro* and *in vivo*. Differentiation capabilities were restored when re-expressing a wild-type form of the phosphatase, while catalytically deficient PTPMT1 or truncated mutant lacking mitochondrial localization failed to do it. PTPMT1 deficiency altered mitochondrial metabolism by disrupting phosphatidylinositol phosphate (PIP) homeostasis leading to an enhanced fatty acid-induced activation of mitochondrial uncoupling protein 2 (UCP2) (Yu et al., 2013). UCP2 has also been reported to tightly control the fate of human pluripotent stem cells (hPSCs) by preventing mitochondrial glucose oxidation and promoting glycolysis (Zhang et al., 2011). Indeed, in hPSCs, UCP2 shunts substrates such as pyruvate from glucose oxidation, promoting glycolysis and nucleotide biosynthesis through the pentose phosphate pathway. Suppression of the UCP2-dependent glucose supply during hPSC differentiation reduced glycolytic capacity and contributed to the transition into oxidative metabolism and lineage specification (Zhang et al., 2011) (**Figure 1**).

Importantly, a recent study reported that human pluripotent embryonic stem cells (ESCs) could dynamically modulate their glycolytic metabolism in response to changes in the culture conditions or during the conversion between different states (i.e., from primed hESCs to either naive hESCs or differentiated cells), while maintaining pluripotency capacities (Gu et al., 2016). By using metabolic flux analyses, the authors observed that naive hPSCs displayed both elevated glycolysis rates and mitochondrial OXPHOS. This process, driven at least partly by c-Myc oncogene, would allow naive PSCs to switch to one or the other metabolic route according to the demand or the microenvironment (Gu et al., 2016). hPSCs grown as feeder-free cultures were also more sensitive to the inhibition of monocarboxylate transporter MCT1, a target of Myc-driven glycolysis.

The metabolic shift from glycolysis to OXPHOS when cells are differentiating is definitely not an exclusive mechanism. Indeed, a study reported that ESCs could utilize glycolysis-derived pyruvate not to fuel TCA cycle and OXPHOS but to increase the pool of cytosolic acetyl-CoA, through mitochondrial citrate export, in order to support histone acetylation required for pluripotency (Moussaieff et al., 2015). Importantly, in the intestinal crypt, mitochondrial oxidative phosphorylation and glycolysis concomitantly occur in intestinal stem cells [ISCs; Lgr5^+^ crypt base columnar cells (CBCs)] and adjacent differentiated Paneth cells, respectively (Rodriguez-Colman et al., 2017). Indeed, a metabolic symbiosis between lactate-generating Paneth cells and lactate-consuming Lgr5^+^ CBCs allows to sustain mitochondrial oxidative phosphorylation in the latter while maintaining stem cell function and crypt homeostasis. It is also noteworthy that some other adult stem cells, like muscle stem cells (satellite cells), localize to aerobic niches at the vicinity of capillary vessels and mostly rely on OXPHOS, mainly through mitochondrial fatty acid and pyruvate oxidation (Ryall et al., 2015). Moreover, a metabolic switch toward glycolysis, linked to epigenetic reprogramming, occurs when those satellite cells progress toward more committed states (Ryall et al., 2015).

A switch from OXPHOS to glycolysis has also been reported during the conversion of differentiated cells into induced pluripotent stem cells (iPSCs) (Folmes et al., 2011; Shyh-Chang et al., 2013a). The upregulation of glycolysis eventually occurs early during iPSC reprogramming, prior to the re-expression of pluripotency markers (Hansson et al., 2012; Shyh-Chang et al., 2013a), indicating that the exacerbated glycolytic pathway is not necessarily associated to a pluripotent state, rather representing the preferred metabolic state to sustain high proliferation rate. Moreover, cell proliferation enhances iPSC reprogramming stochastically (Hanna et al., 2009), making the link between glycolysis, proliferation and pluripotency even more complex. Promotion of glycolysis enhances iPSC reprogramming, whereas pharmacological inhibition of glycolysis or stimulation of OXPHOS impairs iPSC reprogramming (Yoshida et al., 2009; Zhu et al., 2010; Folmes et al., 2011). Similarly, mouse ESCs show increased activity in the pentose phosphate pathway that allows rapid nucleotide synthesis (Filosa et al., 2003; Varum et al., 2011; Manganelli et al., 2012).

### Mitochondrial Pyruvate Import in Stem Cells

**FIGURE 2:**
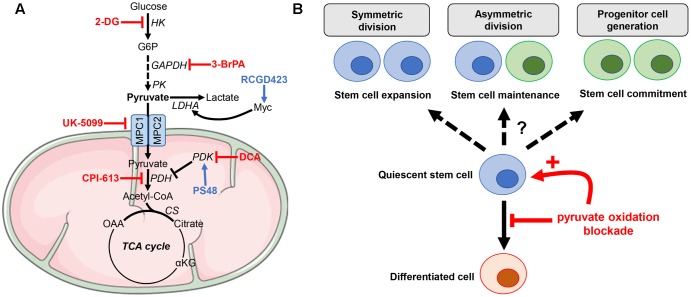
Manipulating pyruvate metabolism in stem cells. **(A)** Small molecules targeting either reductive or oxidative metabolism of pyruvate can be used to rewire stem cell metabolism. **(B)** Blockade of pyruvate oxidative metabolism may impair cell differentiation while promoting stem cell activation toward either expansion, maintenance or commitment. 2-DG, 2-deoxyglucose; 3-BrPA, 3-bromopyruvate; DCA, dichloroacetate.

Mitochondrial pyruvate import serves as the junction between cytoplasmic glycolysis and mitochondrial oxidative phosphorylation. This transport toward the mitochondrial matrix is mediated through the mitochondrial pyruvate carrier (MPC), a 150-kDa complex formed by MPC1 and MPC2 subunits located on the inner mitochondrial membrane (Bricker et al., 2012; Herzig et al., 2012). Both *MPC1* and *MPC2* genes (also known as *BRP44L* and *BRP44*, respectively) are weakly expressed in ESCs and their expression increases during differentiation [e.g., into cardiomyocytes, *MPC1*, *p* = 4.8E-6 and *MPC2 p* = 6.3E-5 (Cao et al., 2008) or into neural cells (*MPC1*, *p* < 0.0001; *MPC2*, *p* = 0.0061) (Fathi et al., 2011)]. The defect of mitochondrial pyruvate uptake in ESCs, associated with an increase in lactate generation and attenuation of mitochondrial activity, actively participates for the maintenance of stem cell populations (Schieke et al., 2008; Mandal et al., 2011). Conversely, enhancement of pyruvate oxidation may alter stemness capacities by hampering several supportive biosynthetic pathways, including the pentose phosphate pathway and one-carbon metabolism (Filosa et al., 2003; Varum et al., 2011; Manganelli et al., 2012; Shyh-Chang et al., 2013b). Recent studies provided new insights on the role of MPC in stem cell function and the regulation of the balance between self-renewal and differentiation (Flores et al., 2017; Schell et al., 2017). In proliferating Lgr5^+^ ISCs, located at the base of the intestinal crypt, genetic deletion of *MPC1* increased proliferation and expanded the stem cell compartment (Schell et al., 2017). The same phenotype was observed in intestinal organoids treated with UK-5099, a potent specific MPC blocker (**Figure 2A**), which inhibits the transporter by specifically modifying a thiol group on the carrier (Halestrap, 1975, 1976). Finally, the authors also showed that MPC knock-out (KO) in ISCs from Drosophila increased their proliferation while MPC overexpression inhibited it Schell et al. (2017). A second study reported that MPC1 deletion in hair follicle stem cells (HFSCs) accelerated their activation and the hair cycle by increasing lactate production while LDHA KO induced the opposite phenotype (Flores et al., 2017). Interestingly, the authors observed similar activating effects in HFSCs when inhibiting MPC1 transporter with UK-5099 compound. Of note, this study also reported the role of Myc in HFSCs since treatment with RCGD423, a Myc activator (**Figure 2A**), increased lactate production and promoted stem cell activation (Flores et al., 2017).

In cancer cells, a low level of MPC protein expression has been correlated with poor survival in several cancer types including lung, colon, kidney clear cell, prostate and esophagus squamous cell carcinomas (Schell et al., 2014; Zhong et al., 2015; Wang et al., 2016; Li Y. et al., 2017). Moreover, some studies reported that genetic deletion or pharmacological inhibition of MPC could induce the expression of stem cell markers in prostate, ovarian, and colon cancers and led to the development of more aggressive tumors (Schell et al., 2014; Zhong et al., 2015; Li X. et al., 2017). Indeed, MPC1-KO murine prostate cancer cells showed a metabolic reprogramming toward aerobic glycolysis and glutamine anaplerosis with reduced ATP production (Li X. et al., 2017). This phenotype was associated with enhanced migratory capacities and resistance to both chemotherapy and radiotherapy. In addition, MPC1-KO cells expressed significantly higher levels of stemness markers, such as Nanog, HIF-1α, Notch1, CD44, and ALDH (Li X. et al., 2017). Other investigators also reported that overexpression of MPC1 and MPC2 in colorectal cancer cell lines decreased cell growth in spheroids and reduced tumor size in subcutaneous xenografts. Additionally, expression of the stem cell markers ALDH, Lin28A, Lgr5, and Nanog was suppressed in the MPC-overexpressing tumor cells (Schell et al., 2014).

Further investigations are however required to identify the mechanism(s) underlying the link between stemness, MPC expression and tumor progression. Indeed, some studies have reported that the MPC inhibitor UK-5099 could hamper tumor growth, when administered in combination with BPTES or etomoxir (which inhibit glutamine or fatty acid metabolism, respectively) (Vacanti et al., 2014; Yang et al., 2014) and it could reduce pyruvate carboxylase-dependent anaplerosis associated with breast cancer-derived lung metastases (Christen et al., 2016).

### Mitochondrial Pyruvate Metabolism in Stem Cells

After mitochondrial import, pyruvate can be either converted into acetyl-CoA through PDH activity or into oxaloacetate *via* the pyruvate carboxylase (PC)-dependent anaplerotic reaction (**Figure 1**). ^13^C-based metabolomic studies recently unraveled that PC activity was increased in astrocytes compared to NSCs (Palm et al., 2015; Sa et al., 2017). Indeed, PC contributed for up to 81% of the pyruvate pool entering the TCA cycle in astrocytes, making this enzyme a key mediator of the metabolic rewiring accompanying astrocytic lineage specification from NSCs (Sa et al., 2017). Dichloroacetate (DCA), a PDK inhibitor that mediates PDH de-repression (**Figure 2A**), redirects pyruvate from lactate to acetyl-CoA and promotes the pluripotency of ESCs, without affecting their viability (Moussaieff et al., 2015). This observation is in contrast with a previous study reporting that DCA blocks iPSC reprogramming (Folmes et al., 2011). Another study reported that PS48, a PDK1 activator that inhibits the entrance of pyruvate into the mitochondria, enhanced reprogramming efficiency for human primary somatic cells (Zhu et al., 2010) (**Figure 2A**). Although ALT and ME activities can also contribute to pyruvate homeostasis in the mitochondria, to the best of our knowledge, there is no study reporting a specific role for these enzymes in stem cell maintenance. Finally, it should be stressed that although the blockade of pyruvate oxidative metabolism impairs differentiation of most stem cell populations, the outcomes of such treatment on the fate of stem cells (i.e., expansion, maintenance, or commitment) may vary according to the tissue origin and/or microenvironmental factors (**Figure 2B**). This caveat does not allow to propose a unifying model that would link metabolic preferences and stem cell maintenance/activation. It also highlights the need to test therapeutic strategies aiming to influence stem cell differentiation in adequate experimental setups.

### Cancer Stem Cells, Pyruvate Metabolism, and Treatment Resistance

Increasing evidence for the existence of self-renewing [CSCs, also referred to as tumor-initiating cells (TICs)] has led to the emergence of an important new area of cancer research (Reya et al., 2001; Visvader and Lindeman, 2008; Beck and Blanpain, 2013). By analogy to normal stem cells, CSCs were defined as a subset of cancer cells able to self-renew and to generate all the differentiated cells found within a tumor (Singh et al., 2004). CSCs have been more frequently reported in some cancer types including leukemia (Lapidot et al., 1994; Uckun et al., 1995; Bonnet and Dick, 1997), brain tumors (Singh et al., 2004), colorectal cancer (Dalerba et al., 2007; O’Brien et al., 2007; Ricci-Vitiani et al., 2007), and breast cancer (Al-Hajj et al., 2003). They are thought to actively participate to the recurrence of tumors, after initially successful chemotherapy and/or radiation therapy. For example, in KRAS^G12D^-mutant pancreatic tumors, genetic deletion of the KRAS^G12D^ allele leads to a dramatic tumor regression, but a small subset of resilient cells resists and eventually mediates tumor relapse (Viale et al., 2014). These surviving cells display features of CSCs and importantly rely on OXPHOS for survival (unlike the tumor bulk which is mainly glycolytic). Inhibitors of oxidative metabolism impaired tumor relapse when KRAS^G12D^ was re-expressed in this model (Viale et al., 2014). Of note, CSCs of human pancreatic cancer-derived xenografts were also found to rely on OXPHOS (Sancho et al., 2015). Moreover, quiescent human leukemia stem cells, responsible for tumor resistance, could be targeted through their high dependency to oxidative phosphorylation, in a BCL-2-dependent manner (Lagadinou et al., 2013). Recently, a study showed that therapy-resistant chronic myeloid leukemia stem cells mostly rely on upregulated oxidative metabolism for their survival (Kuntz et al., 2017). Combinatory treatment with imatinib and tigecycline, a FDA-approved mitochondrial translation inhibitor, was able to overcome resistance by eradicating the stem cell-enriched populations (CD34^+^ and CD34^+^CD38^-^). Nevertheless, when comparing the metabolic phenotype of CSCs vs. non-CSCs populations, the tumor type, the biological model (2D vs. 3D cultures) or the microenvironmental conditions may determine whether CSCs will favor glycolysis or OXPHOS as preferred energy source. Glioma stem cells (GSCs) rely on OXPHOS to a much larger extent than differentiated glioma cells but can easily switch toward the use of glycolysis when mitochondrial respiration is impaired, to maintain energy production and cell survival (Vlashi et al., 2011). Another study reported that nutrient-restricted conditions contribute to brain tumor progression by selecting brain TICs that upregulate high-affinity glucose transporter GLUT3 and are thereby endowed with a competitive advantage for survival and proliferation (Flavahan et al., 2013).

Metabolic reprogramming in CSCs has also been associated with their increased pro-metastatic potential through the induction of an epithelial-to-mesenchymal transition (EMT). In particular, distinct metabolic programs were shown to be associated with the preferential metastatic sites for primary tumors. Liver-metastatic breast cancer cells exhibit a unique metabolic program compared to bone- or lung-metastatic cells, characterized by increased conversion of glucose-derived pyruvate into lactate and a concomitant reduction in glutamine and OXPHOS metabolism (Dupuy et al., 2015). This metabolic adaptation was supported by an increased HIF-1α activity and expression of PDK1. The latter was required for promoting liver metastasis, by helping tumor cells to survive under nutrient limitation and hypoxia. Another study reported that the transcriptional factor Snail repressed fructose-1,6-bisphosphate (*FBP1*) gene expression, resulting in the induction of glycolysis in basal-like breast cancer cells undergoing EMT (Dong et al., 2013). Loss of FBP1 was also associated with an inhibition of OXPHOS and ROS production. This metabolic rewiring enhanced CSC-like features and promoted tumorigenicity of breast cancer cells, making the loss of FBP1 a critical oncogenic event in EMT and metastatic potential of breast cancer cells. Finally, EMT and cancer stemness were also recently reported to be linked in pancreatic cancer models (Krebs et al., 2017). By using genetic mouse models, investigators showed that the transcription factor Zeb1 (a well-known EMT-inducer) led to the formation of precursor lesions, invasion and metastasis in distant organs. Genetic invalidation of Zeb1 impaired the metabolic plasticity of tumor cells by inhibiting both OXPHOS and glycolytic capacities. Altogether, these studies provide indisputable insights supporting a direct link between cancer stemness, metastatic potential and metabolic reprogramming.

## Conclusion

Metabolic reprogramming is necessary for regulating the fate of stem cell populations. It is now acknowledged that stem cells use a wide variety of substrates, such as glucose, glutamine, and fatty acids to support production of biosynthetic intermediates and/or energy during proliferation or differentiation. Recent findings reviewed here point to pyruvate as one of the key metabolites controlling stem cell function. The metabolic fate of pyruvate, toward lactate production or mitochondrial metabolism, is a key aspect of the regulation of stem cell compartment (or pool), as it participates to the decision between the maintenance of self-renewal or the promotion of clonal expansion and differentiation. Importantly, drugs regulating pyruvate metabolism were reported to alter the balance between self-renewal and differentiation states (**Figure 2B**), proving that controlling the pool of stem cells in normal tissues and tumors will soon be an achievable clinical goal.

## Author Contributions

The author confirms being the sole contributor of this work and approved it for publication.

## Conflict of Interest Statement

The author declares that the research was conducted in the absence of any commercial or financial relationships that could be construed as a potential conflict of interest.
